# Analysis of the efficacy of lenalidomide in patients with intermediate-1 risk myelodysplastic syndrome without 5q deletion

**DOI:** 10.3892/etm.2013.1218

**Published:** 2013-07-11

**Authors:** YAN YANG, SUJUN GAO, HONGQIONG FAN, HAI LIN, WEI LI, JUAN WANG

**Affiliations:** 1 Department of Haematology and Oncology, The First Hospital of Jilin University, Changchun, Jilin 130021, P.R. China; 2 Department of Oncology, People’s Hospital of Jilin Province, Changchun, Jilin 130021, P.R. China

**Keywords:** lenalidomide, myelodysplastic syndrome

## Abstract

The aim of this study was to evaluate the efficacy and adverse effects of lenalidomide in the treatment of intermediate-1 risk non-5q deletion [non-del (5q)] myelodysplastic syndrome (MDS). A total of 30 patients with MDS were classified through G-banding chromosome karyotype analysis and fluorescence *in situ* hybridization (FISH). According to the International Prognostic Scoring System scores, among the 30 patients, 23 and seven cases had scores of 0.5 and 1.0, respectively. Lenalidomide (Revlimid^®)^, 10 mg/day) was administered for 21 days every 28 days. All 30 cases were treated with lenalidomide for at least three cycles, including 20 cases with four cycles. The patients did not require erythropoietin, cyclosporine or iron chelation treatments. Statistical analysis was performed using SPSS statistical software version 13.0, and comparisons among groups were conducted using a t-test. The efficacy of lenalidomide was demonstrated in patients with intermediate-1 risk non-del (5q) MDS. Peripheral blood cell counts were improved following treatment, and absolute neutrophil, haemoglobin and platelet counts increased following 2–4 cycles of treatment. All patients became stable having undergone three cycles of treatment; however, 17 patients with chromosomal abnormalities had no cytogenetic response to the treatment, as confirmed through the FISH test. Patients with intermediate-1 risk non-del (5q) MDS treated with lenalidomide did not achieve complete haematological remission, although they demonstrated haematological improvement.

## Introduction

Myelodysplastic syndrome (MDS) is a heterogeneous clonal haematopoietic stem cell disorder characterised by ineffective haematopoiesis and a high risk of progression to acute myeloid leukemia (AML). The ineffective haematopoiesis results in one or more peripheral cytopaenias, dysplasic changes in the haematopoietic cell lines and typical cytogenetic abnormalities (1). Conventional drugs, including haematopoietic cytokines and chemotherapy drugs, have shown little efficacy in the treatment of the disease, although in recent years, numerous new drugs have been listed or have been subject to clinical trials. Three drugs have recently been approved by the US Food and Drug Administration (FDA) for the treatment of MDS within the last seven years: lenalidomide, azacytidine and decitabine. Lenalidomide is predominantly used for the treatment of patients with MDS with 5q deletion [del (5q)] cytogenetic abnormalities. However, the 2009 version of the National Comprehensive Cancer Network (NCCN) guidelines also recommended lenalidomide for the management of low/intermediate-1 risk MDS.

A number of studies have demonstrated that lenalidomide treatment is effective in patients with MDS with del (5q), and may increase the number of peripheral blood cells, thus enabling patients to undergo a reduced number of infusions, and improving the quality of life (2–4). However, there have been few investigations into the efficacy of lenalidomide for patients with intermediate-1 risk MDS without del (5q), particularly amongst Chinese individuals. Since March 2009, the treatment of patients with low/intermediate-1 risk MDS at the Department of Haematology and Oncology of The First Hospital of Jilin University (Changchun, China) has included the administration of lenalidomide. By March 2011, 30 patients had received this treatment. The clinical data obtained from the patients are summarised in the current study.

## Patients and methods

### Patients

A total of 30 patients in the Department of Haematology and Oncology of The First Hospital of Jilin University were diagnosed with MDS through peripheral blood counts, bone marrow smears, bone marrow biopsies and cytogenetic analyses. This study was conducted in accordance with the Declaration of Helsinki and with approval from the Ethics Committee of the First Hospital of Jilin University. Written informed consent was obtained from all participants. All patients were classified through G-banding chromosome karyotype analysis combined with a fluorescence *in situ* hybridization (FISH) test (+8, −5/5q-, −7/7q-, 20q-, −Y and −12/12p- were selected as the cytogenetic probes in the FISH method). Karyotype anomalies were found in 11 cases, including seven with 20q-, three with +8, one with dup (1) (q22 31), and four cases that failed in the karyotype analysis. The FISH test revealed anomalies in 17 cases, including six with +8, seven with 20q-, four with 12p- and 13 cases with negative results. The patients included 14 males and 16 females, with ages ranging from 13 to 83 years old (median age, 52 years). The duration between diagnosis and treatment ranged from 0 to 9 months (mean, 4.1 months). Six cases of refractory anemia (RA), one case of refractory thrombocytopaenia (RT), one case of refractory anemia with ring sideroblasts (RARS), 17 cases of refractory cytopaenia with multilineage dysplasia (RCMD) and five cases of MDS-unclassified (MDS-U) were identified according to the World Health Organization (WHO) classification system (5). In addition, the International Prognostic Scoring System (IPSS) was used evaluate the 30 patients, and of these, 23 had a score of 0.5 and seven had a score of 1.0 (5). The average erythropoietin (EPO) levels were >500 mU/ml in all the patients; in addition, two out of 27 cases tested positive for human leukocyte antigen (HLA)-DR15.

### Management

Lenalidomide (Revlimid^®^, 10 mg/day) was administered for 21 days every 28 days. All 30 cases were treated with at least three cycles, with 20 cases being treated with four cycles. Red blood cell transfusion was given to those with symptomatic anaemia, and platelet transfusions were given to those with platelet counts of <6×10^9^/l or those with obvious thrombocytopenic bleeding. None of the patients required EPO, cyclosporine (CSA) or iron chelation treatments.

### Evaluation

Peripheral blood cell counts were monitored every 3–5 days, whereas liver and renal functions were reviewed every two weeks. The bone marrow morphology, cytogenetic analysis and FISH test were performed once the patients had undergone three cycles of treatment.

### Statistical analysis

Statistical data were processed using SPSS statistical software version 13.0 (SPSS, Inc., Chicago, IL, USA), and comparisons among groups were conducted using a t-test. P<0.05 was considered to indicate a statistically significant result.

## Results

### Changes in peripheral blood cell counts in the 30 patients following treatment

The efficacy of lenalidomide as a treatment for patients with intermediate-1 risk non-del (5q) MDS was demonstrated by the peripheral blood cell counts of the patients showing improvement following lenalidomide treatment. A total of 2–4 cycles of treatment resulted in the absolute neutrophil, haemoglobin and platelet counts of the patients increasing; however, the changes were not statistically significant (P>0.05). Analysis within the subgroups showed that the differences in the peripheral blood cell counts between the patients with scores of 0.5 and a duration of ≤3 months between diagnosis and treatment and the patients with scores of 1.0 and a duration of >3 months between diagnosis and treatment were statistically significant (P<0.05). Platelet recovery in the former subgroup was more rapid and marked, with the fastest platelet recovery of 20q- observed in seven patients. However, patients with initial platelet counts of <10×10^9^/l and haemoglobin levels of <45 g/l demonstrated a poor response to the treatment (Tables II and III, respectively).

### Evaluation of the bone marrow morphology and cytogenetic analysis

All patients became stable following three cycles of treatment; however, 17 cases with chromosomal abnormalities demonstrated no cytogenetic response, as confirmed by the FISH test.

### Adverse events

In the first two weeks, 40% of the patients treated with lenalidomide exhibited mild rashes (12 cases); however, the majority of them recovered without any treatment. One case was treated with glucocorticoids and an HT-3 receptor antagonist. Within one week, 11 patients (36.67%) who had initially shown abdominal distension experienced an improvement in the symptoms with continuous lenalidomide treatment. Following three cycles of treatment, one patient suffered from deep venous thrombosis (DVT) in the left lower limb, and although urokinase was administered, the thrombosis was not completely recanalised. In addition, all patients presented with liver and kidney dysfunctions.

## Discussion

Lenalidomide exhibits immunological and biological properties, extending to cytokine modulation, T-cell costimulation and angiogenesis inhibition (6–8). Therefore, the effects of the drug on the MDS clone and the microenvironment may contribute to the therapeutic action. These effects include, but are not limited to, suppression of tumor necrosis factor-α and the induction of other inflammatory cytokines, such as interleukins-1β, 6, 8 and 12; costimulation of the T-cell-specific immune response; expansion of natural killer cells and enhancement of cytotoxicity; suppression of the endothelial response to angiogenic molecules; downregulation of cell-adhesion molecules; potentiation of EPO-receptor signaling and modification of lineage commitment. Furthermore, lenalidomide has been demonstrated to promote cell cycle arrest and display direct antineoplastic activity in a variety of cancer cell lines and primary specimens. Although a precise cellular target has not been identified to date, recent investigations have indicated that lenalidomide acts through the inhibition of phosphatase activity in the common deleted region (CDR) of 5q, which is important in cell cycle regulation through a defect in ribosomal protein function. As such, lenalidomide acts through direct cytotoxic mechanisms in patients with the del (5q) cytogenetic abnormality, in addition to affecting the bone marrow microenvironment in patients without this lesion (by suppressing the effects of proapoptotic and proinflammatory cytokines) (4,9–11).

The beneficial results of lenalidomide have been particularly evident for patients with del (5q) chromosome abnormalities (10,12,13). A randomised clinical trial demonstrated that lenalidomide treatment may have been effective in improving the health-related quality of life (HRQL) outcomes of the tested patients (14). Lenalidomide has recently been approved by the FDA for the treatment of patients with low-risk MDS, particularly for patients with del (5q)(15). For patients without del (5q), however, the effects of lenalidomide remain unclear, and its potential use and activity in non-del (5q) MDS require further investigation (16,17). A phase II study evaluated lenalidomide efficacy in 214 transfusion-dependent patients with low or intermediate-1 risk MDS without del (5q). The results showed that 26% of the non-del (5q) patients achieved transfusion independence following a median of 4.8 weeks of treatment (13). In a study by Sibon *et al*(18), 31 consecutive patients with lower-risk non-del (5q) MDS and anemia refractory to erythropoiesis-stimulating agents (ESA) were treated with lenalidomide in a compassionate programme. A total of 15 patients (48%) demonstrated an erythroid response, including 10 of the 27 (37%) previously transfusion-dependent (RBC-TD) patients who became transfusion-independent (RBC-TI). Out of these 15 patients, nine experienced a relapse, while the remaining six (40%) were still responding and transfusion-free for at least 11(+)–31(+) months subsequently (median time, 24 months). There was a lower erythroid response rate in patients with refractory cytopaenia with multilineage dysplasia (27% versus 60%); in addition, the response rate tended to be higher in patients treated with lenalidomide in combination with ESA (55% versus 36%). The response duration was significantly longer in patients who became RBC-TI and in patients treated with lenalidomide following primary resistance to ESA. It was concluded that lenalidomide was an interesting second-line therapeutic option and that the combination of lenalidomide with ESAs in the context of the study required further investigation (18).

The present study aimed to observe the efficacy of lenalidomide in patients with intermediate-1 risk non-del (5q) MDS. It was observed that lenalidomide did not result in complete haematological remission in the patients, although it improved the peripheral blood cell counts in the patients with intermediate-1 risk MDS. Platelet recovery was more marked in the patients with IPSS scores of 0.5 and with a duration of ≤3 months between diagnosis and treatment. The majority of patients achieved platelet transfusion independence, i.e. the safety level of non-spontaneous bleeding (≥20×10^9^/l). Furthermore, certain patients achieved red blood cell transfusion independence (≥60 g/l) following treatment. Patients with initial platelet and haemoglobin counts of <10×10^9^/l and <45 g/l, respectively, demonstrated a poor response to the treatment. Patients with a duration of <3 months between diagnosis and treatment showed marked improvements in their blood cell counts, whereas those with a duration of >3 months between diagnosis and treatment demonstrated no apparent improvement. Therefore, lenalidomide is not a recommended treatment for patients with severe cytopaenia and with a prolonged duration between diagnosis and treatment.

At present, two phase III studies on lenalidomide are underway. One of these is known as the AZA-004 trial, which studies the effect of lenalidomide on the transformation of MDS into AML (19). The other study is examining the efficacy of lenalidomide in patients with non-del (5q) intermediate-1 risk MDS. It is expected that satisfactory results will be obtained from these two clinical trials.

The reported rate of DVT (VTE) for patients with MDS receiving lenalidomide during the first 2 years of post-marketing exposure has been revealed to be low (0.53%). Disproportionality analysis demonstrated a statistically meaningful correlation between VTE and lenalidomide concomitantly used with ESAs in patients with MDS; however, the correlation was not statistically significant when lenalidomide was used in the absence of ESAs (20). Of the 30 patients examined in the current study, one patient suffered from DVT in the left lower limb following three cycles of treatment, and although urokinase was administered, the thrombosis was not completely recanalised. This result was not consistent with those presented in other studies, and more cases are required to make further observations.

In conclusion, lenalidomide has demonstrated efficacy in patients with intermediate-1 risk non-del (5q) MDS, and is indicated to be a safe form of treatment.

## Figures and Tables

**Table I:** Clinical data of 30 patients with MDS.

Case no.	Gender (M/F)	Age (years)	Time from diagnosis to treatment (months)	WHO classification	Chromosome karyotype	FISH result	IPSS score
1	F	13	0	MDS-U	47XX,+8[9]/46XX[11]	+8	1.0
2	M	31	4	RA	46,XY[20]	12p^−^	0.5
3	M	35	2	RA	46,XY[20]	12p^−^	0.5
4	F	37	2	RCMD	46,XX[20]	Negative	0.5
5	F	40	4	RCMD	46,XX,del(20)(q^11^) [6]/46,XX[14]	20q^−^	0.5
6	F	42	3	RCMD	46,XX,del(20)(q^11^) [5]/46,XX[15]	20q^−^	0.5
7	M	45	6	RCMD	Failure	+8	1.0
8	F	46	3	RCMD	46,XX,del(20)(q^13^) [7]/46,XX[13]	20q^−^	0.5
9	M	50	3	RCMD	46,XX[20]	Negative	0.5
10	M	51	2	RA	46,XY,dup(1)(q^22–31^)[8]/46,XY[12]	Negative	0.5
11	F	51	1	RCMD	46,XX[20]	Negative	0.5
12	F	51	3	RCMD	47,XX,+8[7]/46XX[13]	+8	1.0
13	M	52	6	MDS-U	46,XY,del(20)(q^13^) [5]/46,XY[15]	20q^−^	0.5
14	M	52	7	RCMD	46,XY,del(20)(q^11^) [7]/46,XY[13]	20q^−^	0.5
15	M	52	8	RCMD	46,XY[20]	12p^−^	1.0
16	F	52	2	RCMD	Failure	Negative	0.5
17	F	52	5	RA	46XX[15]	Negative	0.5
18	M	53	4	RCMD	46,XY[20]	Negative	0.5
19	F	53	6	MDS-U	46,XX,del(20)(q^11^) [7]/46,XY[13]	20q^−^	0.5
20	M	54	7	RT	46XY[20]	12p^−^	0.5
21	F	54	8	RCMD	Failure	+8	1.0
22	M	56	9	RCMD	46,XY[20]	Negative	0.5
23	F	57	2	MDS-U	46,XX,del(20)(q^11^) [12]/46,XY[8]	20q^−^	0.5
24	M	59	2	RARS	46,XY[20]	Negative	0.5
25	M	60	1	RCMD	46,XY[20]	Negative	0.5
26	F	62	3	MDS-U	47,XX,+8[5]/46XX[15]	+8	1.0
27	F	65	2	RCMD	46,XX[20]	Negative	0.5
28	F	66	6	RCMD	Failure	+8	1.0
29	F	69	9	RA	46,XX[20]	Negative	0.5
30	M	83	3	RA	46,XY[20]	Negative	0.5

MDS, myelodysplastic syndrome; M, male; F, female; WHO, World Health Organization; FISH, fluorescence *in situ* hybridization; IPSS, International Prognostic Scoring System; MDS-U, MDS-unclassified; RA, refractory anemia; RCMD, refractory cytopaenia with multilineage dysplasia; RT, refractory thrombocytopaenia; RARS, refractory anemia with ring sideroblasts.

**Table II:** Changes in peripheral blood cell counts following treatment (n=30).

		Following treatment	
		
Peripheral blood cell counts	Prior to treatment	2 cycles	4 cycles
Absolute neutrophil count (×10^9^/l)	0.95±0.47	1.02±0.59	1.28±0.84
Haemoglobin (g/l)	59.1±16.1	61.6±16.8	68.6±14.8
Platelet count (×10^9^/l)	30.7±67.8	40.2±18.8	50.1±85.6

Results are presented as the mean ± 2 standard deviations.

**Table III:** Changes in platelet count following treatment in the subgroups.

Subgroup	n	Count prior to treatment (×10^9^/l)	Count following 3 cycles (×10^9^/l)	P-value
Time from diagnosis to treatment ≤3 months; IPSS=0.5	14	31.4±29.6	58.6±25.8	<0.05
Time from diagnosis to treatment >3 months; IPSS=1.0	16	26.5±48.6	31.7±68.3	>0.05

Results are presented as the mean ± 2 standard deviations. IPSS, International Prognostic Scoring System.
